# Accelerated Recovery of Consciousness after General Anesthesia Is Associated with Increased Functional Brain Connectivity in the High-Gamma Bandwidth

**DOI:** 10.3389/fnsys.2017.00016

**Published:** 2017-03-24

**Authors:** Duan Li, Viviane S. Hambrecht-Wiedbusch, George A. Mashour

**Affiliations:** ^1^Department of Anesthesiology, University of MichiganAnn Arbor, MI, USA; ^2^Center for Consciousness Science, University of MichiganAnn Arbor, MI, USA; ^3^Neuroscience Graduate Program, University of MichiganAnn Arbor, MI, USA

**Keywords:** consciousness, general anesthesia, ketamine, functional connectivity, coherence, transfer entropy, gamma, electroencephalogram

## Abstract

Recent data from our laboratory demonstrate that high-frequency gamma connectivity across the cortex is present during consciousness and depressed during unconsciousness. However, these data were derived from static and well-defined states of arousal rather than during transitions that would suggest functional relevance. We also recently found that subanesthetic ketamine administered during isoflurane anesthesia accelerates recovery upon discontinuation of the primary anesthetic and increases gamma power during emergence. In the current study we re-analyzed electroencephalogram (EEG) data to test the hypothesis that functional cortical connectivity between anterior and posterior cortical regions would be increased during accelerated recovery induced by ketamine when compared to saline-treated controls. Rodents were instrumented with intracranial EEG electrodes and general anesthesia was induced with isoflurane anesthesia. After 37.5 min of continuous isoflurane anesthesia, a subanesthetic dose of ketamine (25 mg/kg intraperitoneal) was administered, with evidence of a 44% reduction in emergence time. In this study, we analyzed gamma and theta coherence (measure of undirected functional connectivity) and normalized symbolic transfer entropy (measure of directed functional connectivity) between frontal and parietal cortices during various levels of consciousness, with a focus on emergence from isoflurane anesthesia. During accelerated emergence in the ketamine-treated group, there was increased frontal-parietal coherence {*p* = 0.005, 0.05–0.23 [95% confidence interval (CI)]} and normalized symbolic transfer entropy [frontal to parietal: *p* < 0.001, 0.010–0.026 (95% CI); parietal to frontal: *p* < 0.001, 0.009–0.025 (95% CI)] in high-frequency gamma bandwidth as compared with the saline-treated group. Surrogates of cortical information exchange in high-frequency gamma are increased in association with accelerated recovery from anesthesia. This finding adds evidence suggesting a functional significance of high-gamma information transfer in consciousness.

## Introduction

There is convergent evidence from multiple studies involving multiple species, multiple neuroimaging modalities, multiple analytic techniques, and diverse drug classes suggesting that anesthetic-induced unconsciousness is characterized by a functional fragmentation of cortical and thalamocortical networks (for review see Hudetz and Mashour, 2016). This reversible fragmentation is broadly supportive of the cognitive unbinding theory of anesthetic-induced unconsciousness (Mashour, 2013) as well as the integrated information theory of consciousness (Tononi et al., 2016) and other higher-order perspectives on the neurobiology of subjective experience. In particular, a functional disconnection of the prefrontal cortex from more posterior cortical areas has been consistently found across multiple and diverse anesthetics (Boveroux et al., 2010; Jordan et al., 2013; Lee et al., 2013; Palanca et al., 2015; Bonhomme et al., 2016; Ranft et al., 2016). In line with these findings, recent work in rodents from our laboratory identified directed frontal-parietal connectivity in the high-gamma bandwidth as a neurophysiological finding associated with conscious states that is suppressed during both general anesthesia (propofol, sevoflurane) and sleep (slow-wave, rapid-eye-movement; Pal et al., 2016).

One limitation to studies of brain connectivity is that they are often static measurements in well-defined states, providing little evidence of functional relationships with state transitions. For example, comparisons of connectivity during differential trajectories of anesthetic induction or recovery have not been conducted. We recently demonstrated that subanesthetic doses of ketamine administered during isoflurane anesthesia induced burst suppression, but accelerated emergence compared to saline administration (Hambrecht-Wiedbusch et al., 2017). Ketamine-treated animals also had increased cholinergic tone in the prefrontal cortex and increased gamma power after isoflurane was discontinued. However, the role of enhanced connectivity during the dynamic process of emergence was not assessed. We have previously shown that ketamine, when administered as a sole agent, can increase high-gamma coherence at subanesthetic levels (Pal et al., 2015). In this study, we tested the hypothesis that subanesthetic ketamine administration during isoflurane anesthesia would differentially enhance gamma connectivity during the process of accelerated emergence. We re-analyzed electroencephalogram (EEG) data from our prior study of isoflurane and ketamine (Hambrecht-Wiedbusch et al., 2017) using measures of coherence and normalized symbolic transfer entropy to assess, respectively, functional and directed connectivity.

## Materials and methods

### Experimental design and data recording

As noted, we re-analyzed EEG data from a previously reported study (for full methods, see Hambrecht-Wiedbusch et al., 2017). All experiments were approved by the University of Michigan Committee on Use and Care of Animals and were conducted in accordance with The Public Health Service Policy on Humane Use and Care of Laboratory Animals (National Institutes of Health Publication 80–23). Adult (2–3 month old), male Sprague-Dawley rats (*n* = 20, Harlan/Envigo, Indianapolis, IN) were randomly divided into ketamine and saline groups. At least one week before the experiment, animals underwent head cap surgery. For the purposes of this surgery, general anesthesia was induced with 4% isoflurane (Piramal Critical Care, Inc., Bethlehem, PA), and then reduced to 2.5%. Rats received a single dose of the non-steroidal anti-inflammatory drug carprofen (5 mg/kg, subcutaneous; Rimadyl®, Zoetis Inc., Kalamazoo, MI). Craniotomy was then performed and six electrodes made out of stainless steel wire (A-M Systems, Inc., Carlsborg, WA) were placed at the following stereotaxic coordinates to record EEG: one frontal at 3.0 mm anterior to bregma and 2.5 lateral to the midline (either on the left or right side of the brain, the opposite side of the CMA/11 guide cannula, which was implanted above the prefrontal cortex to measure acetylcholine release, not reported in the current study), two parietal at 4 mm posterior to bregma and 2.5 mm lateral to midline on both sides, two occipital at 8 mm posterior to bregma and 2.5 mm lateral to midline on both sides and one on the nasal commissure as the reference electrode. In addition, rats had two electromyography electrodes placed into the neck muscle to evaluate muscle movement during emergence. Animals were then allowed to recover from surgery for 7 days.

**Figure 1:**
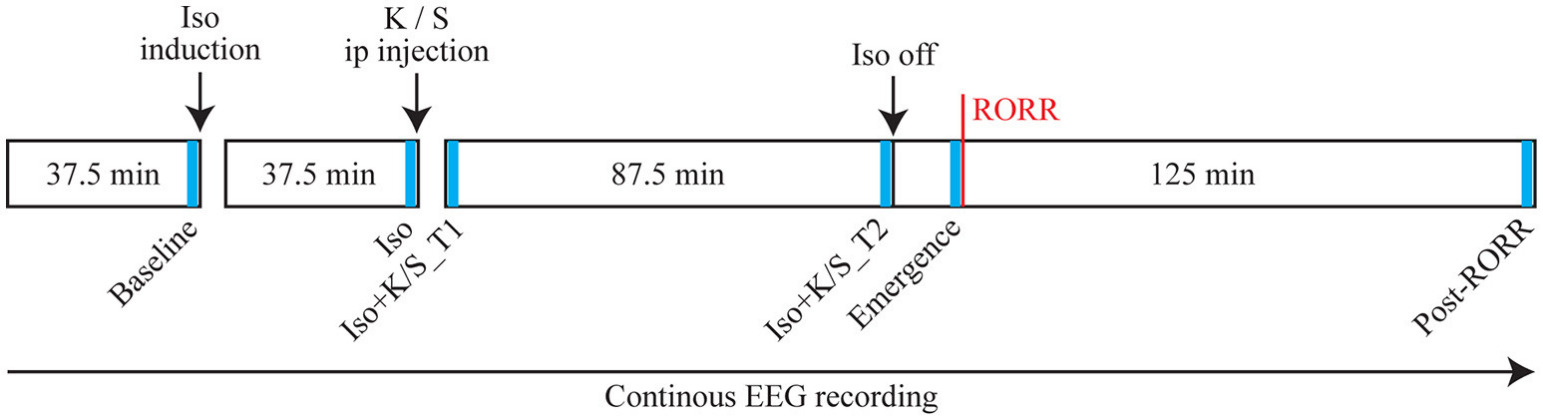
**Schematic time line illustrating the procedure for each animal in this study**. Text above the boxes describes the manipulations performed and text in the boxes illustrates the time line. The red vertical line indicates the time of return of righting reflex (RORR). The electroencephalogram (EEG) was recorded throughout the entire experiment, from which six 2-min segments were selected at specific time points (as indicated in blue bars) for statistical comparisons. Iso: Isoflurane; K: Ketamine; S: Saline; ip: intraperitoneal.

Figure 1 illustrates the experimental design. On the day of the experiment, animals were connected to the EEG recording system, and placed into a modified Raturn. The EEG signal was continuously recorded during the entire experiment. After the baseline wake phase, the Raturn was sealed and filled with isoflurane in high-flow oxygen (10 L/min) until the in- and outlet of the Raturn was reading 2.5% isoflurane for 2 min. After anesthetic induction, as defined by the loss of righting reflex, the animal was placed on its back and a rectal temperature probe was inserted through a door in the Raturn. Isoflurane levels were then kept at 1.5% throughout the rest of the experiment. After 37.5 min of exposure to isoflurane at a stable level, the Raturn door was opened and 25 mg/kg ketamine or an equivalent volume of saline was injected into the intraperitoneal (ip) space of the animal. This is subanesthetic dosing of ketamine for rats, which lose the righting reflex at 150 mg/kg ip (Pal et al., 2015). After stable isoflurane levels returned, 87.5 min of EEG data were collected after ketamine or saline injection, then isoflurane was discontinued and the time it took for return of righting reflex (RORR; the surrogate for emergence time) was measured during the recovery period (with recording for 125 min). Emergence time was determined by two independent observers, at least one of whom was blinded to the experimental condition.

EEG signals from each rat were amplified (x 5000) and filtered (0.1–300 Hz, Grass amplifier Model 15 LT, 15A54 Quad Amplifier, Warwick, RI). Data were digitized and recorded at a sampling rate of 1,000 Hz using AcqKnowledge software (V4.1, MP150, Biopac Systems, Inc, Goleta, CA). The raw EEG signals from ipsilateral frontal and parietal channels were exported into MATLAB (version 2015a; MathWorks, Inc., Natick, MA) and downsampled to 500 Hz (resample.m function in Matlab signal processing toolbox) for further analysis. By visual inspection and spectral analysis of the EEG signals, one ketamine-treated rat and three saline-treated rats were excluded because of bad signal quality. Thus, the sample sizes in subsequent analysis were *N* = 9 for ketamine and *N* = 7 for saline-treated rats.

### Coherence analysis

The functional connectivity between frontal and parietal channels was measured by the magnitude squared coherence method (mscohere.m function in Matlab signal processing toolbox). Specifically, the two time series of 10-s were divided into 2-s sub-windows with 80% overlap, each sub-window was multiplied with a Hamming window, and the coherence was estimated from the cross-spectra and auto-spectra of two series using Welch's averaged, modified periodogram method. To mitigate the potential bias of coherence, a series (*N* = 20) of surrogate data were generated by randomly shuffling one of the two signals, while keeping the other signal unchanged (Canolty et al., 2006); the coherence was calculated with these shuffled data, the mean of which was subtracted from the original coherence as the final estimation of functional connectivity (Papana et al., 2011).

The frontal-parietal coherence estimate was calculated at each 10-s window pair and each frequency bin over the frequency range of 0.5–250 Hz, then averaged at the following frequency bands: theta (4–10 Hz), low-gamma (25–50 Hz), med-gamma (70–110 Hz), high-gamma (110–160 Hz). We chose these general bandwidths because of their relevance to arousal states based on our recent study of neural correlates of wakefulness in rats (Pal et al., 2016); the specific frequency bandwidths of gamma differ somewhat because of unique gamma increases due to ketamine. The emergence time and the duration time after RORR were variable among rats. Emergence time [in median and 95% confidence interval (CI)] was 470 (402–574) s for ketamine-treated rats and 730 (579–1,081) s for saline-treated rats. The duration time after RORR was 7,110 (7,000–7,186) s for ketamine-treated rats and 6,830 (6,476–6,989) s for saline-treated rats. These durations were rescaled to the median time for each group, in order to obtain the group-level temporal plots by taking the average across rats.

As shown in Figure 1, six 2-min epochs were selected as follows: (1) Baseline: the last two min of baseline phase of wakefulness; (2) Iso: the last two min of isoflurane phase; (3) Iso + K/S_T1: the first two min of the isoflurane after injection phase; (4) Iso + K/S_T2: the last two min of the isoflurane after injection phase; (5) Emergence: the last two min before RORR; (6) Post_RORR: the last two min of the recording after RORR. The averaged coherence values were calculated over the six studied epochs for each rat for statistical comparisons.

### Analysis of directed connectivity

We used normalized symbolic transfer entropy (NSTE) to assess directed connectivity. NSTE is an information-theoretic measure, and our previous studies have validated its use to measure cortical connectivity changes in humans (Lee et al., 2013) and rats (Borjigin et al., 2013; Pal et al., 2016). Briefly, TE measures the amount of information provided by the additional knowledge from the past of the source signal in the model describing the information between the past and the future of the target signal (Schreiber, 2000), and the basic principle of STE is to analyze amplitude orders instead of amplitude values with the advantage of faster computation and improved robustness to noise (Staniek and Lehnertz, 2008). NSTE is normalized STE, with the potential bias of STE removed by concomitant analysis of surrogate data (Papana et al., 2011), and then divided by the entropy within the target signal (Lee et al., 2013).

In the calculation of NSTE, three parameters are required: embedding dimension (*d*_*E*_), time delay (τ), and transfer time (δ). We filtered the frontal and parietal EEG signals into four indicated frequency bands (theta, low-, mid-, and high-gamma) and segmented the filtered data into non-overlapped 10-sec windows. For all windows, we followed previous studies (Borjigin et al., 2013; Lee et al., 2013) and fixed the embedding dimension *d*_*E*_ = 3. As the time delay τ defines a broad frequency-specific window of sensitivity for NSTE (Jordan et al., 2013; Sitt et al., 2014; Ranft et al., 2016), we used τ = 25, 5, 2, and 1, corresponding to theta, low-, mid- and high-gamma respectively; for each window, we searched the transfer time δ = 1–50 (corresponding to 2–100 ms) and selected the one that yielded maximum feedback and feedforward NSTE, respectively. The group-level temporal plots of frontal-parietal feedback and feedforward NSTE were computed by taking the median across rats. For statistical comparisons, the averaged connectivity values were calculated over the six studied periods for each rat.

### Statistical analysis

Statistical analyses were conducted in consultation with the Center for Statistical Consultation and Research at the University of Michigan. All EEG-derived frontal-parietal coherence and feedback/feedforward connectivity values were exported to IBM SPSS Statistics version 24.0 for Windows (IBM Corp. Armonk, NY) and GraphPad Prism version 7.00 for Windows (GraphPad Software, Inc., La Jolla, CA). The descriptive statistics (mean ± *SD*) were estimated and plotted. Statistical comparisons were analyzed using linear mixed models (LMM), to test (1) the difference between ketamine-treated (*n* = 9) and saline-treated (*n* = 7) rats, (2) the changes between the six studied epochs within the ketamine- or saline-treated groups, and (3) the difference between feedback and feedforward connectivity. LMM analysis offers more flexibility in dealing with unequal numbers of rats and allowed us to take into account the variability between rats by including a random effect associated with the intercept for each rat. For the model of coherence values, the fixed effects included group, studied epoch, and the interaction between them. For the models of directed connectivity, the fixed effects included group, studied epoch, direction, and the interaction between every pair of factors. We used restricted maximum likelihood estimation and diagonal covariance structure with heterogeneous variances and zero correlation between elements. For all statistical comparisons, the uncorrected *p*-value and the 95% CI of the difference were reported. A *p* < 0.05 was considered statistically significant. To enhance readability of the figures, only significant levels are provided, while the exact *p*-value and 95% CI are provided in a tabular format in the supplementary material.

## Results

### Effect of ketamine on frontal-parietal coherence during isoflurane anesthesia and recovery

**Figure 2:**
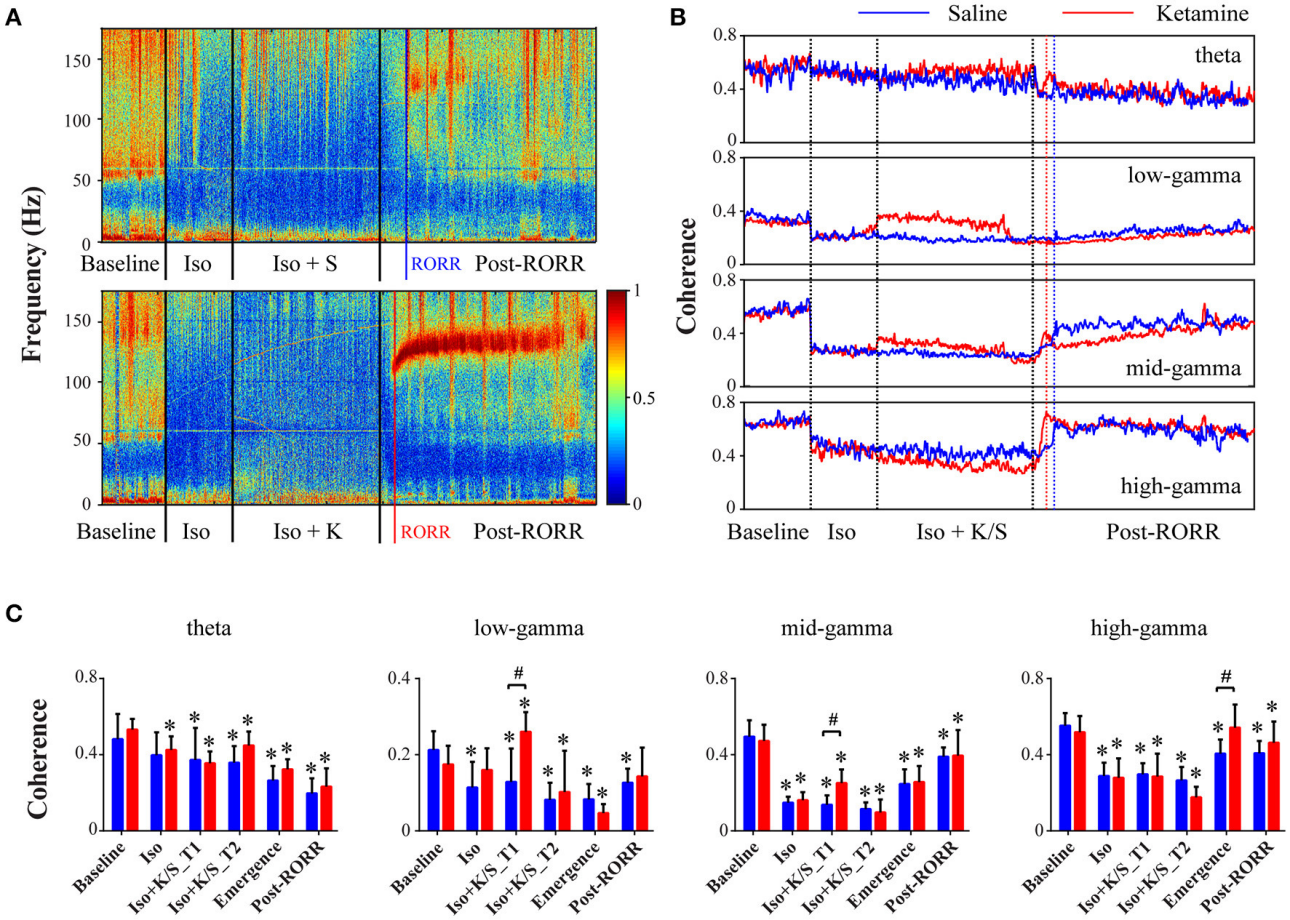
**Effect of subanesthetic ketamine on frontal-parietal coherence. (A)** Representative cohereogram with saline (top panel) or ketamine (bottom panel) administration during isoflurane anesthesia, measured in non-overlapped 10-s bins. The black vertical lines indicate the start and endpoint of the different phases of wake, isoflurane, isoflurane after injection, and recovery; the blue (or red) vertical line marks the time of RORR for the saline (or ketamine)-treated rat. **(B)** Group-level temporal changes of theta and gamma coherence with saline-treated (*n* = 7, blue line) and ketamine-treated (*n* = 9, red line) rats, smoothed over 1-min window. The emergence time and the duration time after RORR were rescaled to the median time across saline- and ketamine-treated rats. **(C)** The mean and *SD* (saline: blue bars; ketamine: red bars) of theta and gamma coherence at the six studied epochs. Significant changes as compared to baseline are indicated using an asterisk (^*^) over the data for ketamine and saline group, whereas significant differences between two groups at each of six stages was marked by a pound sign (#), using linear mixed model analysis (uncorrected *p* < 0.05).

Isoflurane anesthesia induced a significant depression of frontal-parietal coherence across all frequency bands (Figures 2A,B). For saline-treated rats, the mid- and high-gamma (70–160 Hz) coherence returned at emergence (Figures 2B,C, Table S1), which is consistent with a previous study of propofol and sevoflurane anesthesia (Pal et al., 2016). The injection of ketamine during isoflurane anesthesia caused immediate increases in low- and mid-gamma (25–110 Hz) coherence (Figures 2B,C, Table S2), which may be related to the significantly increased burst-suppression ratio that was previously reported (Hambrecht-Wiedbusch et al., 2017). When compared to saline-treated rats during emergence, ketamine-treated rats showed a dramatic increase in high-gamma coherence [*p* = 0.005, 0.05–0.23 (95% CI)] (Figures 2A,C, Table S2). Both ketamine- and saline-treated rats achieved the highest level of high-gamma coherence at emergence, while mid-gamma coherence showed gradual increases after RORR, and theta coherence remained at low levels during recovery as compared to baseline (Figures 2B,C). The changes in frontal-parietal coherence were not consistently associated with changes in frontal or parietal power (see Figure S1 in the supplementary material).

### Effect of ketamine on frontal-parietal directed connectivity during isoflurane anesthesia and recovery

**Figure 3:**
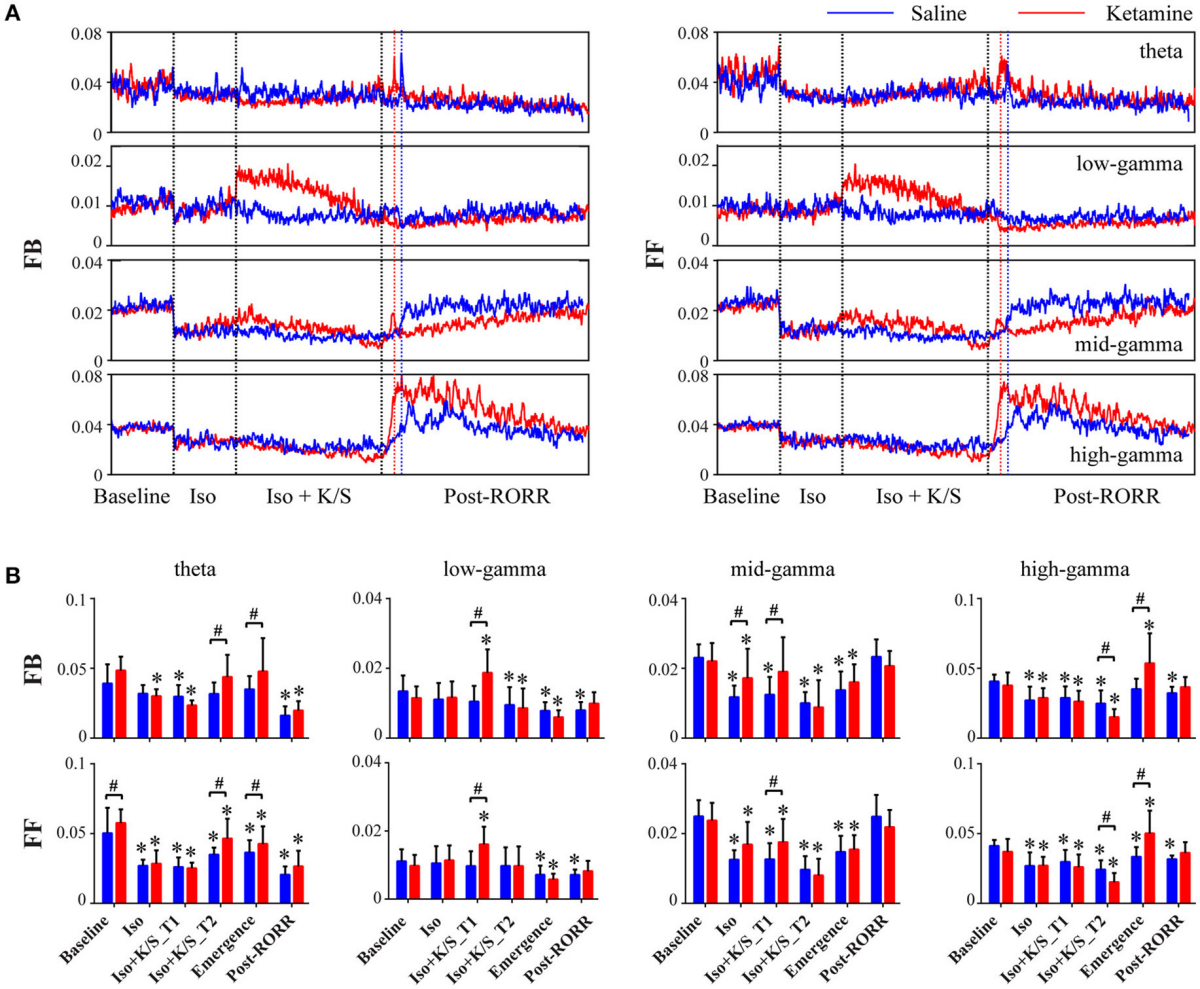
**Effect of subanesthetic ketamine on frontal-parietal directed connectivity. (A)** Group-level temporal changes of feedback (FB, frontal to parietal) and feedforward (FF, parietal to frontal) connectivity at theta, low-, mid-, and high-gamma bands in saline-treated (*n* = 7, blue line) and ketamine-treated (*n* = 9, red line) rats, measured in non-overlapped 10-s bins and smoothed over 1-min window. The black vertical lines indicate the start and endpoint of the different phases of wake, isoflurane, isoflurane after injection, and recovery, and the blue (or red) vertical line marks the time of RORR for the saline (or ketamine)-treated rat. The emergence time and the duration time after RORR were rescaled to the median time across saline- and ketamine-treated rats. **(B)** The mean and *SD* (saline: blue bars; ketamine: red bars) of theta and gamma connectivity at the six studied epochs. Significant changes as compared to baseline are indicated using an asterisk (^*^) over the data for ketamine and saline group, whereas significant differences between two groups at each of six stages are marked by a pound sign (#), using linear mixed model analysis (uncorrected *p* < 0.05).

Isoflurane anesthesia decreased feedback and feedforward connectivity at theta, mid-, and high-gamma bands, which returned at emergence for saline-treated rats (Figure 3, Table S3). The injection of ketamine during isoflurane anesthesia caused increases in low- and mid-gamma connectivity (Figure 3, Table S4). In parallel with frontal-parietal coherence, ketamine-treated rats showed significant increases in high-gamma connectivity at emergence as compared to saline-treated rats [feedback: *p* < 0.001, 0.010–0.026 (95% CI); feedforward: *p* < 0.001, 0.009–0.025 (95% CI)]; there was no significant difference between feedback and feedforward connectivity in either ketamine- or saline-treated rats [feedback vs. feedforward: *p* = 0.23, −0.004–0.006 (95% CI) for ketamine-treated rats; *p* = 0.40, −0.003–0.008 (95% CI) for saline-treated rats]. In addition, ketamine-treated rats showed increased theta feedback and feedforward connectivity at emergence [feedback: *p* = 0.03, 0.001–0.018 (95% CI); feedforward: *p* = 0.02, 0.001–0.018 (95% CI)] and even before the discontinuation of isoflurane [feedback: *p* = 0.006, 0.003–0.020 (95% CI); feedforward: *p* = 0.005, 0.004–0.020 (95% CI)]. Consistent with frontal-parietal coherence findings, both ketamine- and saline-treated rats achieved the highest level of high-gamma connectivity at emergence, while mid-gamma connectivity showed gradual increases after RORR, and theta connectivity remained at low levels during recovery as compared to baseline (Figure 3).

**Figure 4:**
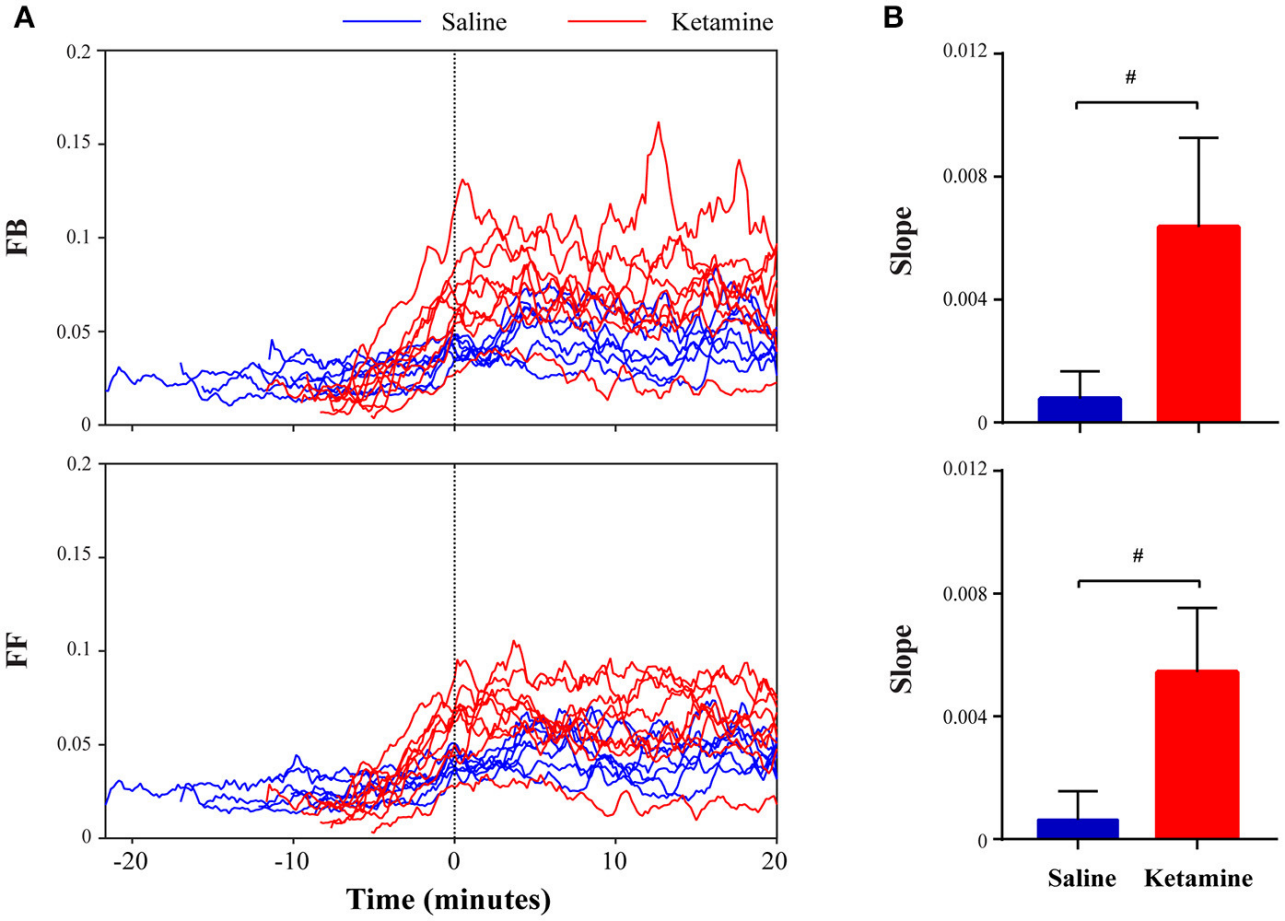
**Ketamine administration induces increased high-frequency connectivity during emergence. (A)** Temporal evolution of high-gamma feedback and feedforward connectivity from discontinuation of isoflurane anesthesia to 20 min after return of righting reflex (RORR) in individual rats (saline: blue lines; ketamine: red lines). Time 0 corresponds to the time of RORR. **(B)** The slope of high-gamma feedback and feedforward connectivity vs. time during emergence, as estimated by fitting a linear regression model for an individual rat. The data are shown in mean and *SD*, and significant differences between saline- and ketamine-treated rats are marked by a pound sign (#), using the Mann-Whitney *U*-test (*p* < 0.05).

To further demonstrate the effect of subanesthetic ketamine on high-gamma connectivity during emergence, Figure 4 shows the temporal progression of high-gamma feedback and feedforward connectivity from the discontinuation of isoflurane anesthesia to 20 min after RORR in individual rats. Ketamine-treated rats showed dramatic increases in high-gamma connectivity during emergence (Figure 4A), with the slope of high-gamma connectivity vs. time, as estimated by fitting a linear regression model, significantly higher than that in saline-treated rats (feedback: 0.0064 ± 0.0029 vs. 0.0008 ± 0.0009, *p* = 0.0002; feedforward: 0.0054 ± 0.0021 vs. 0.0006 ± 0.0009, *p* = 0.0003, Mann-Whitney U test; Figure 4B).

## Discussion

The primary finding of this study is that accelerated emergence from general anesthesia is associated with increased corticocortical connectivity. This was confirmed with measures of both functional (coherence) and directional (NSTE) connectivity and was present in both theta and high-gamma bandwidths. We previously demonstrated that theta connectivity (measured by both coherence and NSTE) is elevated during both wakefulness and rapid-eye-movement (REM) sleep (Pal et al., 2016). By contrast, high-gamma connectivity was associated only with states of wakefulness and was disrupted during anesthesia as well as during both slow-wave and REM sleep. This suggests that high-gamma connectivity is associated with connected consciousness whereas theta connectivity is associated with both connected and disconnected consciousness (the latter occurring during REM-mediated dream states). The fact that high-gamma connectivity was found to be at its nadir during REM sleep, when the threshold for arousal is highest, strengthens its association with connected consciousness. However, this previous study was limited because high-gamma connectivity was identified during stable states of consciousness long after emergence that might be confounded by the return of attentional or other cognitive processes (Koch et al., 2016) after full restoration of the neurochemical milieu. In fact, this might be the reason that, in the current study, theta coherence and connectivity remained low after emergence, unlike our prior study that sampled recovery wakefulness more than 1 h after RORR (Pal et al., 2016). The current study adds to the literature by demonstrating a rising slope of high-gamma connectivity (shown in Figure 4) during a dynamic phase leading to recovery, adding evidence for a functional role of high-gamma connectivity in the emergence of consciousness. The fact that high-gamma connectivity is most pronounced around the time of emergence, then gradually decreases thereafter, might relate to network-level effects contributing to the “ignition” required to emerge from the anesthetized state but that are not required to maintain consciousness.

Gamma activity and synchrony have long been explored for a possible role in consciousness (Engel and Singer, 2001). Seminal work in the 1990s suggested that long-range synchronization of 40 Hz activity (followed by rapid desynchronization) was a solution to the problem of how the brain can effectively integrate the activity of discrete neuronal subpopulations to form a unified experience of the world (Rodriguez et al., 1999). However, more recently, 40 Hz activity has been found to persist or even to be augmented during anesthetized states, suggesting that it is not sufficient to sustain consciousness (Imas et al., 2004, 2005; Murphy et al., 2011). It should be noted that persistent synchrony of gamma during general anesthesia is distinct from precise, stimulus-related temporal coordination and is likely inconsistent with the conditions for effective information transfer that appear to be required for normal consciousness. Nonetheless, 40 Hz gamma has fallen out of favor as a correlate of consciousness (Merker, 2013, 2016). By contrast, the power of higher gamma activity, also referred to as high-frequency oscillations and thought to reflect broadband neural spike activity, has been found to be differentially suppressed during general anesthesia. This finding in both humans (Breshears et al., 2010) and rodents (Hudetz et al., 2011) increases the biological plausibility that high-gamma activity and connectivity is at least correlated with states of consciousness.

The methodology of this study is unique because the addition of a second anesthetic drug (ketamine) to a primary inhaled anesthetic (isoflurane) was the causal agent that accelerated recovery and enhanced high-gamma connectivity. The administration of subanesthetic ketamine causes “paradoxical emergence” because it deepens the intra-anesthetic state (as evidenced by burst suppression, a neurophysiologic marker of profound general anesthesia) while reducing the time to emergence once the isoflurane is discontinued (Hambrecht-Wiedbusch et al., 2017). We have previously shown (Pal et al., 2015) that ketamine alone can enhance cortical acetylcholine levels and, at subanesthetic levels just prior to emergence from the anesthetized state, induce a robust increase in high-gamma coherence. Ketamine increases 40 Hz and higher gamma power in humans at both anesthetic (Lee et al., 2013) and subanesthetic (Rivolta et al., 2015; Shaw et al., 2015) doses and might therefore be a tool to enhance gamma activity and communication in the perioperative period or critical care setting.

There are limitations to this study. In terms of the scientific implications, the current methodology does not allow us to distinguish whether high-gamma connectivity is a cause, correlate, prerequisite, or consequence of conscious experience (Aru et al., 2012). Only associations were investigated and the temporal precision in assessing behavioral state transitions in rodents is poor. Other paradigms involving the control of higher gamma activity will enable further insight. Furthermore, only surrogates of communication were assessed; more direct measures of information transfer are required for definitive conclusions (Schroeder et al., 2016). In terms of the clinical implications, we have studied a model that involves anesthesia alone, without the stress and inflammatory burden of surgery or the additional factors of pain and polypharmacy. Whether ketamine has any clinical effect on enhanced recovery or increased functional connectivity must be investigated in a routine clinical care setting.

In conclusion, accelerated recovery of consciousness after general anesthesia is associated with enhanced connectivity in the high-gamma bandwidth. This supports the hypothesis that coherent patterns of high-frequency communication across the cortex are functionally important for consciousness but further investigation is required to establish causal relationships.

## Author contributions

GM conceived of the study; VH performed experiments; DL performed analysis; GM and DL wrote the manuscript; all authors critically reviewed and approved manuscript.

## Funding

Supported by grant R01GM111293 from the National Institutes of Health, Bethesda, Maryland, USA and the Department of Anesthesiology at the University of Michigan, Ann Arbor, Michigan, USA.

### Conflict of interest statement

The authors declare that the research was conducted in the absence of any commercial or financial relationships that could be construed as a potential conflict of interest. The reviewer SM and handling Editor declared their shared affiliation, and the handling Editor states that the process nevertheless met the standards of a fair and objective review.

## Supplementary material

Figure S1 **Effect of subanesthetic ketamine on frontal and parietal power. (A)** Representative spectrogram of frontal (left) and parietal (right) power with saline (top panel) or ketamine (bottom panel) administration during isoflurane anesthesia, measured via Welch's method (pwelch.m function in Matlab signal processing toolbox) in non-overlapped 10-s windows. The black vertical lines indicate the start and endpoint of the different phases of wake, isoflurane, isoflurane after injection, and recovery; the blue (or red) vertical line marks the time of RORR for the saline (or ketamine)-treated rat. **(B)** Group-level temporal changes of theta and gamma power with saline-treated (*n* = 7, blue line) and ketamine-treated (*n* = 9, red line) rats, smoothed over 1-min window. The emergence time and the duration time after RORR were was rescaled to the median time across saline- and ketamine-treated rats.

Table S1 **Statistical comparisons of frontal-parietal coherence at studied epochs as compared to baseline, for saline- and ketamine-treated rats**.

Table S2 **Statistical comparisons of frontal-parietal coherence between saline-treated (*n* = 7) and ketamine-treated (*n* = 9) rats**.

Table S3 **Statistical comparisons of frontal-parietal feedback (FB) and feedforward (FF) connectivity at studied epochs as compared to baseline, for saline- and ketamine-treated rats**.

Table S4 **Statistical comparisons of frontal-parietal feedback (FB) and feedforward (FF) connectivity between saline-treated (*n* = 7) and ketamine-treated (*n* = 9) rats**.
