# Supplementary figures and images for: Accelerated Recovery of Consciousness after General Anesthesia Is Associated with Increased Functional Brain Connectivity in the High-Gamma Bandwidth
Source: Front Syst Neurosci. 2017 Mar 24;11:16. doi: 10.3389/fnsys.2017.00016 (PMC5364164; doi:10.3389/fnsys.2017.00016)

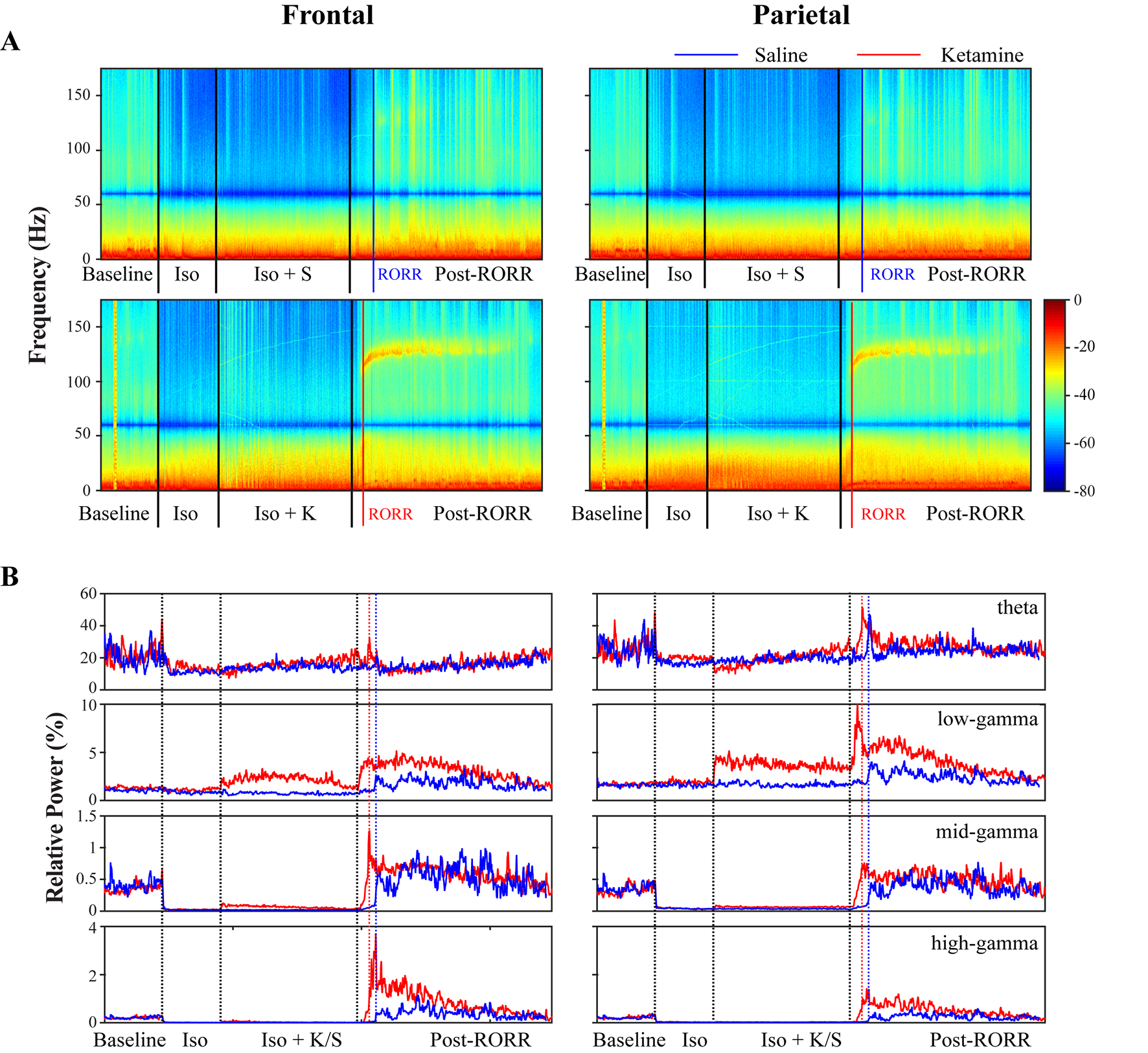

Supplement: Figure S1 — Effect of subanesthetic ketamine on frontal and parietal power. (A) Representative spectrogram of frontal (left) and parietal (right) power with saline (top panel) or ketamine (bottom panel) administration during isoflurane anesthesia, measured via Welch's method (pwelch.m function in Matlab signal processing toolbox) in non-overlapped 10-s windows. The black vertical lines indicate the start and endpoint of the different phases of wake, isoflurane, isoflurane after injection, and recovery; the blue (or red) vertical line marks the time of RORR for the saline (or ketamine)-treated rat. (B) Group-level temporal changes of theta and gamma power with saline-treated (n = 7, blue line) and ketamine-treated (n = 9, red line) rats, smoothed over 1-min window. The emergence time and the duration time after RORR were was rescaled to the median time across saline- and ketamine-treated rats. [file Image1.TIF]
